# Preferential induction of *MLL(Mixed Lineage Leukemia)* rearrangements in human lymphocyte cultures treated with etoposide

**DOI:** 10.1590/S1415-47572009000100022

**Published:** 2009-03-01

**Authors:** María Sol Brassesco, Ana Paula Montaldi, Elza Tiemi Sakamoto-Hojo

**Affiliations:** 1 Departamento de Genética, Faculdade de Medicina de Ribeirão Preto, Universidade de São Paulo, Ribeirão Preto, SPBrazil; 2 Departamento de Puericultura e Pediatria, Faculdade de Medicina de Ribeirão Preto, Universidade de São Paulo, Ribeirão Preto, SPBrazil; 3 Departamento de Biologia, Faculdade de Filosofia Ciências e Letras de Ribeirão Preto, Universidade de São Paulo, Ribeirão Preto, SPBrazil

**Keywords:** etoposide, FISH, *MLL* translocations

## Abstract

Topoisomerase II inhibitors are effective chemotherapeutic agents in the treatment of cancer, in spite of being associated with the development of secondary leukemia. Our purpose was to determine the effects of etoposide on different genomic regions, aiming at discovering whether there are preferential sites which can be targeted by this drug in peripheral lymphocytes from healthy individuals. The *in vitro* treatment with low doses of etoposide (0.25, 0.5, and 1 μg/mL, in 1 hour-pulse or continuous-48 h treatment) induced a significant increase in chromosomal aberrations, detected by conventional staining and FISH with specific probes for chromosomes 8 and 11, compared with untreated controls (p < 0.05). Additionally, the frequencies of alterations at 11q23, detected by *MLL* specific probes, were significantly higher (p < 0.005) in treated cells than in controls. In contrast, an analysis of rearrangements involving the *IGH* gene did not disclose differences between treatments. The present results demonstrated the potential of etoposide to interact with preferential chromosome sites in human lymphocytes, even at concentrations below the mean plasma levels measured in cancer patients. This greater susceptibility to etoposide-induced cleavage may explain the more frequent involvement of *MLL* in treatment-related leukemia.

## Introduction

Etoposide is one of the most effective anticancer drugs frequently used for the treatment of hematological malignancies and solid tumors. However, its use has been associated with the development of secondary leukemia. The main target of this drug is the nuclear enzyme DNA-topoisomerase II that catalyzes topological changes necessary for normal DNA metabolism, including replication, transcription and recombination (Austin and Marsh, 1998). This drug, as well as other topoisomerase II inhibitors, exerts its toxic effects by inhibiting the enzyme function, thereby causing the accumulation of cleavable complexes and introducing high levels of transient protein-associated breaks in the genome of treated cells (Burden and Osheroff, 1998; Hande, 1998).

Therapy related leukemias, associated with topoisomerase II inhibitors, often present rearrangements involving the *MLL* gene on chromosome band 11q23, or to a lesser extent, t(8;21), t(3;21); t(8;16), t(15;17), t(9;22) or inv(16), to then emerge as overt leukemias in a period of 2 to 3 years following therapy completion (Felix, 1998).

The *MLL* gene plays an important role during fetal development and is a critical regulator of *HOX* genes during hematopoiesis. This function is subverted in leukemias through cleavage, recombination and chimeric fusion with more than 50 gene partners (Pais *et al.*, 2005Meyer *et al.*, 2006). However, in contrast to the diversity with which *MLL* translocates, the mapping of breakpoints revealed that most translocations occur within a 8.3 kb *BamH1* fragment, known as the break-cluster region (BCR), located between exons 8 and 14 (Echlin-Bell *et al.*, 2003). According to Strout *et al.*, (1998), the BCR region presents 8 *Alu* repeats, various topoisomerase II consensus sites, 7/8 *X*-like octamers and V(D)J cryptic recognition signals, which might explain the high susceptibility of this gene to damaging agents. Nonetheless, other and subsequent studies have shown that DNA structure properties, such as scaffold associated regions and DNAse I hypersensitive sites, can act as recombination hot spots (Broeker *et al.*, 1996; Strissel *et al.*, 1998; Hensel *et al.*, 2001; Reichel *et al.*, 2001; Strick *et al.*, 2006).

Furthermore, the biological evidence that leukemias in newborns originate *in utero*, has led to the hypothesis that maternal exposure to topoisomerase II inhibitors during pregnancy could be associated with an increased risk of leukemia (Ross, 2000). It is well known that synthetic and natural flavonoids interact with topoisomerase II and form the cleavable complex, in spite of being anticarcinogenic in certain cases (Greaves, 1997). According to Wiemels *et al.* (1999), the exposure of mothers and fetuses to substances that interact with the topoisomerase II inhibitors present in diets, medicines and the environment can in order of magnitude, be lower in terms of dose, when compared to the drugs used in chemotherapy. Nevertheless, in some cases they can be as biologically active as the topoisomerase II inhibitors used in cancer treatment.

Therefore, by considering the more frequent involvement of *MLL* in treatment-related leukemogenesis, we aimed at studying whether low concentrations of etoposide would also preferentially promote 11q23 translocations over rearrangements within other chromosomal locations.

**Figure 1:**
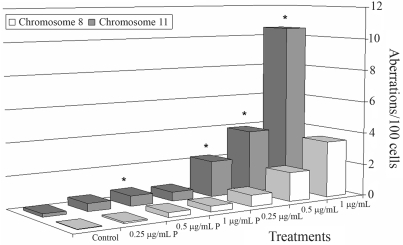
Mean aberration frequencies on chromosomes 8 and 11 (detected by chromosome painting), observed in lymphocyte cultures treated with different etoposide concentrations and negative controls. P: one-hour-pulse treatment.*Statistically different p < 0.05.

**Figure 2:**
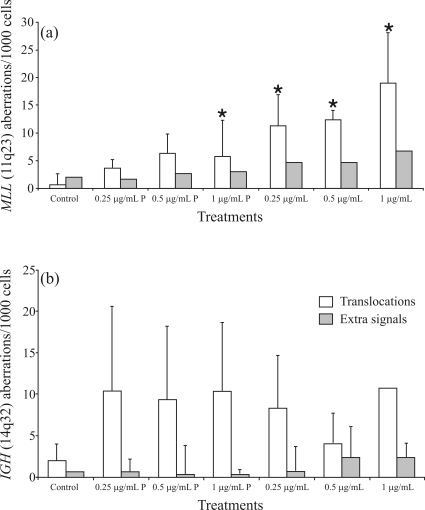
*MLL* (A) and *IGH* (B) mean translocation and extra signal frequencies observed in peripheral blood lymphocytes cultures treated with different etoposide concentrations. P: one-hour-pulse treatment. *statistically different. p < 0.05.

**Figure 3:**
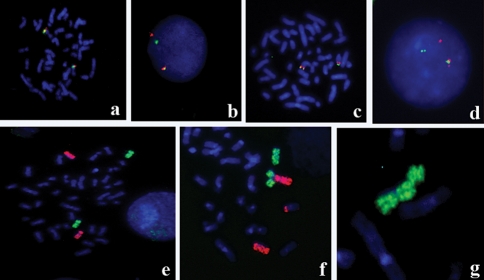
FISH analysis of peripheral lymphocytes treated with etoposide. a) and b) Normal methaphase with two *MLL* copies at 11q23 and translocated nucleus presenting the separation of signals, respectively. c) Normal metaphase with two *IGH* gene copies at 14q32. d) Translocated nucleus presenting the separation of signals. e) Normal metaphase showing chromosome pair 8 (WCP 8 Spectrum-Green) and chromosome pair 11 (WCP 11 Spectrum-orange). f) Partial metaphase showing a triradial chromosome involving both chromosomes. g) Partial metaphase showing a chromatid break. Figures of altered cells correspond to 1 μL/mL etoposide treatment.

**Figure 4:**
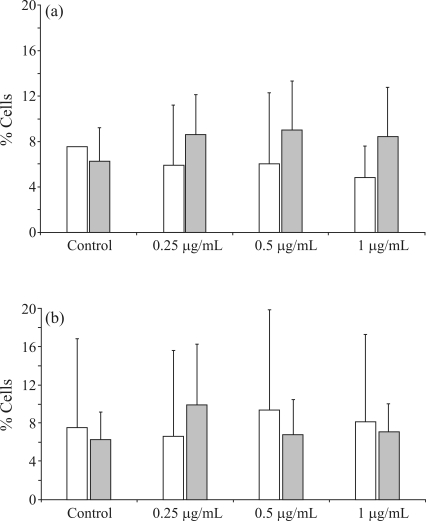
Mean aberration frequencies of apoptotic (white bars) and necrotic (grey bars) cells observed in negative controls and in lymphocyte cultures treated with different etoposide concentrations: A) One-hour-pulse treatment and B) continous 48 h treatment.

## Material and Methods

### Blood culture and metaphase preparation

Whole blood from three healthy donors (21 years old) were collected in a vacutainer containing the anticoagulant heparin, and cultured in a RPMI-1640 medium supplemented with 15% fetal bovine serum, 1% penicillin-streptomycin, 1% l-glutamine (Gibco, Grand Island, NY), and 1% phytohemagglutinin-P (Gibco). Cell cultures were incubated at 37 °C in a 5% CO_2_ moist atmosphere and harvested at 72 h after culture initiation. Twenty four hours after mitogenic stimulation, Etoposide (Nex-Vep, Bristol Myers Squibb) was added and cells were incubated for 48 h at 37 °C in final concentrations of 0.25, 0.5 and 1 μg/mL. One set of cultures was treated for one hour-pulse, being subsequently washed twice in a RPMI 1640 medium, and subcultured in a supplemented medium until harvesting (47 h recovery period). Colcemid (0.1 μg/mL) was added 90 min prior to harvesting time. After hypotonic treatment (0.075 M KCl) for 30 min at 37 °C, cells were fixed three times with methanol: glacial acetic acid (3:1). The fixed cells were then dropped onto glass slides, allowed to air dry and stored at -20 °C, until being used in the FISH technique.

### Fluorescence *in situ* hybridization

FISH was performed using the commercially available probes LSI MLL Break Apart Rearrangement, according to manufacturer's protocol (Vysis, Downers Grove. IL). The Spectrum-Green labeled probe covers a 350 kb centromeric portion of the *MLL* gene breakpoint region, while the Spectrum-Orange-labeled probe covers a 190 kb region telomeric to the BCR. In addition, LSI IGH Dual Color Break Apart Rearrangement probes (Vysis) were used. These probes hybridize with 14q32 and present the same characteristics as the probes for 11q23. In both cases, the pattern of expected signals for a normal nucleus is two green (yellow) orange signals. In cell harboring translocation, the green and orange signals appear separated without the yellow intersection. This strategy allows for the detection of translocations irrespective of the partner involved.

Chromosomal aberrations were also analyzed by using the whole chromosome painting Spectrum-Orange WCP-8 and Spectrum-Green WCP-11 probes (Vysis).Images were captured by using the Axiovision System (Zeiss, Germany).

### Detection of apoptotic cells

Apoptotic cells were recognized by nuclear condensation and fragmentation. Treated cells were centrifuged and incubated for 5 min at 37 °C with bisbenzimide (Hoechst 33342), propidium iodide and fluorescein diacetate (Sigma Chemical Co., St. Louis, U S A). Then, samples were mounted, coverslipped and analyzed by fluorescence microscopy with a triple filter. Cells were scored and categorized according to differential staining: (1) normal: blue nucleus and green cytoplasm, (2) apoptotic: fragmented blue nucleus and green cytoplasm and (3) necrotic: spherical red nucleus. Five hundred nuclei were analyzed per treatment.

### Statistical analysis

Statistical analyses were performed by using SigmaStat (Jandel Scientific, Co) software. All tests were carried out for α = 0.05.

## Results

The clastogenic effects of etoposide on different genomic regions were analyzed in lymphocyte cultures from 3 healthy probands. Cultures were treated with three drug concentrations, 0.25, 0.5 and 1 μg/mL. Etoposide-treated cells presented a reduction in mitotic indexes (calculated for 1000 cells), albeit there were no significant differences among treatments (p > 0.05). Etoposide induced a statistically significant increase in frequencies of chromosome aberration relative to negative controls (p < 0.0005, One Way Repeated Measures ANOVA). On comparing the different treatments and controls, the Bonferroni method showed significant differences for continuous exposure to etoposide (p < 0.05) (Table 1). Cytogenetic analysis by FISH with whole chromosome probes indicated increased frequencies of altered cells and aberrations located at chromosome 11, when treated cultures and controls were compared (p < 0.05, Friedman Repeated Measures ANOVA on Ranks), in both cases (Table 2). Mean numbers of 800 metaphases were analyzed. Alterations in chromosome 8 also showed increased frequencies (p < 0.05). For both chromosomes, the comparison of different treatments against controls performed by the Bonferroni method showed significant differences for continuous exposure to etoposide (p < 0.05). The individual comparison of mean aberration frequencies per treatment calculated for chromosomes 8 and 11 showed statistically higher frequencies for chromosome 11 than for chromosome 8, in some drug concentrations (Figure 1).

The specific induction of translocations at 11q23 was also analyzed by using the *MLL* gene probe (Vysis). *MLL* rearrangements were scored in nuclei presenting a hybridization pattern with clearly distinct separation of signals. Statistical analysis showed significant differences (p < 0.0005, One Way ANOVA) between treated and untreated cultures. In contrast, the analysis of rearrangements involving the *IGH* gene (14q32) did not disclose significant differences between treatments (p > 0.05, One-Way Repeated Measures ANOVA) (Figure 2). Extra signals were also detected by both specific probes, but their frequencies were not significantly increased (p > 0.05) in treated cultures. Hybridization patterns and examples of chromosome aberrations are presented in Figure 3.

It has been suggested that *MLL* aberrations could result from chromatin fragmentation at initial stages of apoptosis. However, treated lymphocytes did not show etoposide-induced apoptosis (p > 0.05). Similarly, frequencies of necrotic cells did not significantly differ (p > 0.05) from controls (Figure 4).

## Discussion

In the present study, we observed a statistically significant increase in the frequencies of chromosome aberration in peripheral lymphocyte cultures treated with low etoposide concentrations. On comparing with controls, this increase proved to be higher in the continuous 48 h treatment. Treated cultures also showed a reduction in mitotic indexes, even though the differences between treatments were not significant. Several studies have demonstrated that cells are more sensitive to etoposide when treated during the S-phase of the cell cycle, although formation of the cleavable complex has been reported to occur at all phases (Berger *et al.*, 1991). It has been suggested that at low concentrations, the cleavable complex could initiate a series of events that would culminate in the elimination of cells through necrosis or apoptosis (Froelich-Ammon and Osheroff, 1995). In the present study, the morphological analysis of cells by differential fluorescent staining did not show significant induction of apoptotic and necrotic cells by different etoposide concentrations.

A direct correspondence between topoisomerase II consensus sequences and the breakpoints described in therapy-related leukemias has been reported (Felix, 1998). Furthermore, rearrangements at 11q23 are highly reproducible in culture under conditions of treatment with topoisomerase II inhibitors (Aplan *et al.*, 1996). There is also evidence that etoposide-metabolites, cathecol and quinone, also induce breaks at *MLL* BCR, as observed for the drug itself (Lovett *et al.*, 2001). The present results demonstrate the clastogenic activity of etoposide, even at low concentrations over short periods, placed in evidence by the induction of aberrations at chromosome 11 and *MLL* gene, as detected by FISH with specific probes. Aplan *et al.* (1996) showed that double-strand breaks can be induced *in vitro* in myeloid lineages with concentrations as low as 3 μmol (1.7 μg/mL), and that such breaks increase with higher doses, with a plateau at concentrations between 30-100 μmol (17-58 μg/mL). These authors also demonstrated that site-specific cleavage did not require continuous treatments, since cells treated for one hour and recovered after 24 h presented specific cleavage at 11q23.

Our results showed differences between treated and untreated cells even at the lowest concentration (etoposide 0.25 μg/mL), ~0.4 μmol, for one-hour pulse. However, the clastogenic effect was more pronounced under conditions of continuous treatments for 48 h, which is the maximum period of drug stability in cultures (Mader *et al.*, 1991). Even though Aplan *et al.* (1996) showed that the induction of aberrations did not require continuous drug exposure, other authors demonstrated that the effects of topoisomerase II inhibitors disappear after drug removal (Binaschi *et al.*, 1990), suggesting that at low concentrations the possibilities of DNA repair seem to be higher.

Regarding aberrations at the *MLL* gene, Moneypenny *et al.* (2006) demonstrated the induction of breaks (assessed by the comet assay) in fetal hematopoietic cells exposed to low concentrations of etoposide (0.3 μg/mL). Furthermore, increased frequencies of *MLL* rearrangements were also detected by FISH after 24 h, 72 h and 7 days following treatment with etoposide 0.3 and 0.6 μg/mL.

The recurrent association of 11q23 translocations with therapy-related leukemias has led to the suggestion that this region might be preferentially damaged by topoisomerase II inhibitors, and consequently, chromosome 11 might present more aberrations compared with other chromosomes. With this in view, we proposed to analyze induced-damage at chromosome 8 (involved to a lesser extent as it is in leukemia-associated translocations) and at the *IGH* gene (14q32), also involved in lymphoid malignancies.

The analysis of chromosome 8 showed a significant increase of chromosomal aberrations in treated cultures relative to controls, in continuous treatment with etoposide (0.5 and 1 μg/mL), but a comparison of mean aberration frequencies calculated for chromosomes 8 and 11 showed significantly higher frequencies for 11 compared to 8. In addition, rearrangements involving the *IGH* gene (14q32) did not significantly (p > 0.05) increase after etoposide treatment, indicating that this gene is not affected to the same extent as the *MLL* gene by induced-chromosome breaks under the same conditions.

Similarly, the non-random induction of aberrations by etoposide was previously reported by Maraschin *et al.* (1990), demonstrating that chromosomes 1, 11 and 17 were more involved in aberrations than chromosomes 4, 5 and X, which were rarely affected. Regarding other chromosomes, Mosesso *et al.*, (1998) showed that the frequencies of aberrations at chromosomes 2, 4 and 8 were 20%-30% higher than those observed for chromosomes 1, 3 and X.

Differences in susceptibility to induced-breaks might be related to certain characteristics of chromosome structure, since R-band rich chromosomes, such as chromosome 11, are more sensitive than G-band rich ones (Maraschin *et al.*, 1990). In CD34^+^ cells treated with etoposide, Libura *et al.* (2005) showed that aberrations at chromosome 11 occur at frequencies twice as high as at chromosome 4, suggesting that even though t(4;11) is the most frequent translocation in some leukemias, there is still a difference concerning susceptibility to etoposide induced-damage. Also, Escobar *et al.* (2007) showed by comet-FISH assays that etoposide produced more DNA damage at 11q23 in relation to the long arm of chromosomes 5, more specifically 5q31, which is often deleted secondary leukemias associated by previous treatment with alkylating agents.

Previous studies on drug clastogenicity performed at the gene level were unable to detect specific damage at several *loci*, including *SCL, ENL, AF6, TCR*β, *TCR*γ and *TCR*δ, in etoposide-treated cells (Aplan *et al.*, 1996). Likewise, Ng *et al.* (2006) reported a higher susceptibility of the *MLL* gene to treatments with etoposide 10-100 μmol compared to the leukemia-associated *RUNX1* and *AF9* genes.

It has been reported that the origin of gene fusions in lymphoid malignancies, such as t(14;18) *IGH/BCL2,* t(11;14) *IGH/CCD1*, t(11;14) *LMO2/TCR* and t(7;9) *TCR/TAL2*, among others, might involve the V(D)J recombination process (responsible for the rearrangements at T-cell receptor and immunoglobulin genes), apparently through the recognition of heptamer-nonamer cryptic sequences (Vega and Medeiros, 2003).

Increased illegitimate recombination frequencies at *TCR* genes have been identified in agricultural workers exposed to pesticides (Lipkowitz *et al.*, 1992) and in patients under chemotherapy (Abdallah *et al.*, 1995). Chen *et al.* (1996) demonstrated that etoposide induces site-specific rearrangements consistent with V(D)J-recombinase, by studying deletions at the *HPRT* gene level. The loss of exons 2 and 3 appeared to have increased in non-lymphoid cells in a concentration-dependent manner under continuous etoposide-treatment.

In our study, the analysis of the *IGH* gene (14q32) demonstrated only a slight increase in the frequency of aberrations, through different treatments for one hour-pulse. Illegitimate V(D)J recombination has also been associated with the origin of *MLL* aberrations, probably due to the presence of consensus signal sequences at BCR. Gu *et al.* (1992) reported cryptic heptamer-nonamer sequences when analyzing breakpoints at *MLL* and *AF4*, involved in t(4;11), suggesting that the V(D)J-recombinase complex could be directly associated with the emergence of translocation. The involvement of this process has also been suggested by Whitmann *et al.*, (2001), who detected illegitimate recognition sequences at the breakpoint junctions between *MLL* intron 6 and *AF9* intron 8 in patients with leukemia harboring t(9;11). In parallel, several studies have demonstrated the induction of *MLL* aberrations after exposure of cells to different apoptotic stimuli (Stanulla *et al.*, 1997; Sim & Liu , 2002; Betti *et al.*, 2003).

However, there is increasing evidence showing that certain properties related to DNA structure, such as scaffold associated regions and DNAse I hypersensitive sites, indeed act as recombination hot spots (Broeker *et al.*, 1996; Strissel *et al.*, 1998; Hensel *et al.*, 2001; Reichel *et al.*, 2001; Strick *et al.*, 2006). Interestingly, Scharf *et al.*, (2007) demonstrated that the etoposide cleavage site at the *MLL* gene resides within a RNA polymerase II binding site.

In childhood leukemia patients, the mean plasma concentration of topoisomerase II inhibitors is 1.7 μmol (1 μg/mL) in chronic treatments (Edick *et al.*, 2003), reaching 25 μmol after 6 h from administration in some treatment schedules. Some controversy exists in the literature regarding dosage and duration of treatment in the development of therapy-related leukemias. According to Megonigal *et al.* (2000), there is a dose-response relationship in patients treated with alkylating agents but this would not be the case for patients treated with epipodophyllotoxins. On the contrary, other authors postulate the existence of a direct leukemogenic effect after etoposide treatment, dependent on the time and dose administered (Dann and Rowe, 2001). There are reports of t-AML development after exposure to few doses of topoisomerase II inhibitors (Blanco *et al.*, 2001).

Although the factors influencing the predisposition of the *MLL* gene to induced damage by topoisomerase II inhibitors are still unknown, the presence of topoisomerase II consensus sites, scaffold-associated regions and other sequences (such as *Alu* repeats, cryptic V(D)J signal and 7/8 *X*-like sequences, among others), suggest that this gene might be more vulnerable to breakage by enzymes, toxic chemicals and metabolites.

Thus, the increase of *MLL* aberrations induced after treatment with low doses of etoposide, as shown in the present study, provides relevant information regarding the potential effects of etoposide in *in vivo* exposure, demonstrating the evident predisposition of this gene to drug-induced chromosome damage, even at concentrations below the limits observed for patients under chemotherapy.

## Figures and Tables

**Table 1:** Mitotic indexes, chromosome aberration distribution (CAs), total CAs and altered metaphases in cultures treated with etoposide. Independent experiments were performed with blood samples from three individuals.

Treatment	Mitotic index (mean ± SD)	Total CAs^#^/100 cells (mean ± SD)	Altered Metaphases/100 cells (mean ± SD)
Control	5.14 ± 2.30	1.58 ± 0.57	1.58 ± 0.57
0.25 μg/mL (1 h)	5.79 ± 2.28	7.3 ± 0.57	6.31 ± 0.57
0.5 μg/mL (1 h)	5.12 ± 1.04	8.14 ± 2.08	6.83 ± 1.73
1 μg/mL (1 h)	4.78 ± 2.34	9.22 ± 3.78	7.36 ± 2.51
0.25 μg/mL (48 h)	2.71 ± 1.04	81.97 ± 13.20*	35.72 ± 11.53*
0.5 μg/mL (48 h)	2.92 ± 1.36	93.77 ± 58.73*	45.88 ± 22.94*
1 μg/mL (48 h)	1.89 ± 0.17	168.76 ± 44.19*	74.01 ± 12.42*

^#^Chromosome aberrations included chromatidic and chromosome gaps. chromatid and chromosome breaks; fragments. double minutes and other aberrations such as rings. dicentrics. triradial. tetraradial. and complex aberrations. *Statistically different. p < 0.05. Modality of treatment is indicated in brackets: 1 h pulse and 48 h continuous treatment.

**Table 2:** Altered metaphases and chromosomal aberrations (CAs) detected with specific probes for chromosomes 8 and 11 in peripheral blood lymphocytes treated with etoposide. 1000 cells were analyzed per treatment/experiment.

Treatment	Altered Metaphases/100 cells (mean±SD)	Total CAs^#^/100 cells (mean±SD)
	Chromosome 11	
Control	2.00 ± 2.00	0.186 ± 0.19
0.25 μg/mL (1 h)	3.03 ± 2.88	0.46 ± 0.18
0.5 μg/mL (1 h)	3.10 ± 7.81	0.34 ± 0.82
1 μg/mL (1 h)	4.04 ± 4.93	0.46 ± 0.46
0.25 μg/mL (48 h)	21.10 ± 9.45*	2.11 ± 0.94*
0.5 μg/mL (48 h)	17.01 ± 9.60*	3.89 ± 0.67*
1 μg/mL (48 h)	48.70 ± 19.13*	9.85 ± 4.21*

	Chromosome 8	
Control	0.33 ± 0.57	0.03 ± 0.00
0.25 μg/mL (1 h)	0.66 ± 0.57	0.08 ± 0.00
0.5 μg/mL (1 h)	2.15 ± 2.08	0.22 ± 0.24
1 μg/mL (1 h)	3.33 ± 2.88	0.22 ± 0.30
0.25 μg/mL (48 h)	6.46 ± 2.08*	0.70 ± 0.16*
0.5 μg/mL (48 h)	15.28 ± 1.52*	1.76 ± 0.55*
1 μg/mL (48 h)	12.29 ± 13.45*	3.28 ± 1.43*

^#^Chromosome aberrations included chromatidic and chromosome gaps. chromatid and chromosome breaks; fragments. double minutes and other aberrations such as rings. dicentrics. triradial. tetraradial. and complex aberrations. *Statistically different. p < 0.05. Modality of treatment is indicated in brackets: 1 h pulse and 48 h continuous treatment.
